# Examining the relationship between burnout and empathy in healthcare professionals: A systematic review

**DOI:** 10.1016/j.burn.2017.06.003

**Published:** 2017-09

**Authors:** Helen Wilkinson, Richard Whittington, Lorraine Perry, Catrin Eames

**Affiliations:** a Institute of Psychology, Health and Society, University of Liverpool, Liverpool, L69 3GB, UK; b Broset Forensic Department, St. Olav’s University Hospital, Trondheim, 7440, Norway; c Department of Mental Health, Norges Teknisk- Naturvitenskapelige Universitet (NTNU), Trondheim, 7491, Norway; d Mersey Care NHS Foundation Trust, Liverpool, L34 1PJ, UK

**Keywords:** Burnout, Empathy, Healthcare staff, Systematic review

## Abstract

**Objective:**

Empathy and burnout are two related yet distinct constructs that are relevant to clinical healthcare staff. The nature of their relationship is uncertain and this review aimed to complete a rigorous, systematic exploration of the literature investigating the relationship between burnout and empathy in healthcare staff.

**Design:**

A systematic review was conducted in accordance with the Preferred Reporting Items for Systematic Reviews and Meta-Analyses (PRISMA) guidance.

**Data sources:**

Search terms (Burnout OR Burn-out OR “Burn out”) AND (Empathy OR Empath*) enabled identification of studies investigating burnout and empathy in healthcare staff, using five electronic data bases (MEDLINE, PsycINFO, CINAHL Plus, PubMed, and SCOPUS). Manual searching amongst reference lists of eligible articles was also completed.

**Review methods:**

Databases were searched for studies published in the English language, from inception to February 2017. Key inclusion criteria were: 1) participants who were nurses or medical professionals, 2) full written manuscript in English, 3) use of the Maslach Burnout Inventory to assess burnout and a standardized outcome measure for empathy, 4) quantitative methodology exclusively.

**Results:**

Ten eligible studies were reviewed. Of those, seven were conducted in countries where English was not the first language. Eight of the studies provided empirical support for a negative relationship between empathy and burnout. One study provided support for a positive relationship between burnout and empathy. One study reported contradictory evidence with positive and negative correlations between different subscales of the empathy and burnout measures. In general, the quality of the studies was assessed to be good. However, some of the studies failed to provide information pertaining to sample size, with the reporting of data less than adequate from one study.

**Conclusions:**

There was consistent evidence for a negative association between burnout and empathy. This review avoided a common English-speaking country bias of some areas of the literature. Given that all of the studies reviewed were cross sectional, further research is necessary to establish causality.

## Highlights

• As with previous research findings relating to the direction of the relationship between burnout and empathy were not unanimous.
• Only one of the ten studies included in the review supported a positive correlation between burnout and empathy.
• Eight studies reported a negative relationship between burnout and empathy.
• Studies included in this review satisfied all of the quality assessment criteria.
• Burnout is supported as a cross cultural construct.

## 1 Introduction

Empathy is a core element of an effective therapeutic relationship (Yu & Kirk, 2009); however it is a subtle concept that is hard to conclusively define. It is often confused with related concepts such as compassion fatigue and sympathy. Burnout is a related but distinct concept (Maslach, 2003), that needs to be distinguished from empathy. Both of these concepts have been cited in the literature as fundamental to quality of healthcare (Brockhouse, Msetfi, Cohen, & Joseph, 2011), and therefore the exact relationship between the two needs to be examined rigorously.

### 1.1 Burnout

Maslach and Jackson (1981) defined burnout as a psychological syndrome involving physical depletion, feelings of helplessness, negative self-concept, and negative attitudes towards work, life, and others. Their conceptualization cited burnout as an internal reaction to external stressors (Adriaenssens, De Gucht, & Maes, 2015). The Maslach Burnout Inventory ([MBI]; Maslach & Jackson, 1981) is referred to as the ‘gold standard’ for measuring burnout in empirical research (Bradham, 2008; Lee & Ashforth, 1990). Lee and Ashforth (1990) comment on how, although Maslach and Jackson's (1981) definition did not have universal agreement it is widely cited in the literature. This is cited in the literature as the most commonly used measure for assessing burnout in human services (Halbesleben & Demerouti, 2005; Lee & Ashforth, 1990). Indeed, a review of the literature demonstrated 90% of studies utilized the MBI as an outcome measure for burnout (Schaufeli & Enzmann, 1998), and it continues to be used more recently (Torres, Areste, Mora, & Soler-Gonzalez, 2015; Walocha, Tomaszewski, Wilczek-Rużyczka1, & Walocha, 2013).

In line with Maslach and Jackson's (1981) definition of burnout, the MBI measures burnout across three dimensions: emotional exhaustion (EE), depersonalization (DP), and personal accomplishment (PA).

EE is defined as a state of emotional and sometimes physical depletion. Those experiencing EE are likely to feel over-extended and unable to offer emotional support to others; Nyatanga (2014) refer to EE as being central and often the most obvious manifestation of the syndrome. DP is conceptualized as an unfeeling and impersonal response towards recipients of one's care Paris and Hoge (2009). This conceptualization has been supported in the literature as clinicians’ development of negative or cynical attitudes towards service user (Baxter, 1992). Lee and Ashforth (1990) discuss how DP can be seen as a defense which serves to protect against unwanted demand, or reduce perceived threat. Therefore it has been associated with psychological strain, and escape as a way of coping. Maslach (2003) defined a reduced sense of PA as involving a negative view of oneself, particularly in relation to one's work with service users.

Whilst the MBI has good reported reliability and validity (Maslach & Jackson, 1981), it has come under some criticism in relation to the wording and scoring of items. All of the DP and EE items are worded negatively and the PA items are worded positively (Demerouti, Bakker, Nachreiner, & Schaufeli, 2001), indicating that this uni-directional wording may have caused artificial clustering of factors (Bouman, te Brake, & Hoogstraten, 2002; Lee & Ashforth, 1990). Additionally researchers have suggested that ‘exhaustion’ should also include cognitive and physical aspects (Pines, Aronson, & Kafry, 1981; Shinn, 1982).

In response to these criticisms other measures have been developed to address these limitations (e.g. Halbesleben & Demerouti, 2005), however, the utilization of this measure within the empirical literature does not compare with that of the MBI (Maslach & Jackson, 1981).

Prevalence of burnout in western countries within the general working population ranges from 13% to 27% (Lindblom, Linton, Fedeli, Bryngelsson, 2006; Norlund et al., 2010). However, healthcare professionals are referred to as being at increased risk of suffering burnout (Bender & Farvolden, 2008; Gelsma et al., 2006; Morse, Salyers, Rollins, Monroe-DeVita, & Pfahler, 2012), compared with non-helping professions.

Prevalence is documented to be as high as 70% worldwide amongst physicians (Lamothe, Boujut, Zenasni, & Sultan, 2014), with 30%–50% of nurses reaching clinical levels of burnout on self-report measures (Aiken, Clarke, Sloane, Sochalski, & Silber, 2002; Gelsema et al., 2006, Poncet et al., 2007). Burnout has been linked to quality of care, with an international study, Poghosyan, Clarke, Finlayson, and Aiken (2010) reporting that higher self-ratings of burnout were associated with lower self-ratings of quality of nurses own care. Similarly Maslach (2003) cites burnout as the principle reason for job attrition within nurses. Burnout is also linked with increased rates of job turnover and stress-related absences (Potter et al., 2010), estimated to cost £450,000 a year per National Health Service (NHS) Trust in the United Kingdom (Wright, 2005). It is not surprising therefore, that burnout has been widely researched in healthcare settings.

### 1.2 Empathy

Empathy, like burnout, has been widely discussed within the context of medical, nursing, and other healthcare professions in relation to its role in therapeutic relationships and quality of care (Brockhouse et al., 2011, Cunico et al., 2012; Smajdor, Stöckl, & Salter, 2011). Theoretically and conceptually, empathy has seen much attention in the philosophical, psychological, and more recently, cognitive neuroscience literature, with varying definitions and conceptualizations (Decety & Lamm, 2006). It is not within the scope of this review to consider all of these definitions; instead, the reader will be guided through the clinically relevant conceptualizations of empathy, its measurement, pertinence to clinical practice, and links with burnout as a construct.

Rogers (1957) termed empathy as the ability of the clinician to sense the service user's private world as if it were their own, without losing the ‘as if’, hypothetical quality. This sense of distancing, or appropriate level of detachment from the service user's emotion, is supported in subsequent definitions offered by Hojat et al. (2002) and Mercer and Reynolds (2002). The common factor amongst these definitions is the suggestion that empathy bridges the gap between self-experience and that of others (Hodges & Klein, 2001). This may be important for clinicians who, through their therapeutic relationships, are required to empathize for long periods with service users experiencing intense and often negative emotions.

Within this context empathy is understood to have four key dimensions: emotive, cognitive, behavioral, and moral (Morse et al., 1992). The emotive and cognitive components relate to clinicians’ abilities to experience and share in another person's feelings, and intellectually identify and understand another person's feelings from an objective stance. The behavioral dimension refers to a clinician's ability to communicate their understanding of another person's perspective. The fourth, moral dimension, was referred to by Morse et al. (1992) as an internal altruistic motivation to be empathic towards others. This dimension was not supported by a subsequent review of the literature by Decety and Jackson (2004). Despite this lack of support, the moral component could be considered relevant when reflecting on the recent exposure of failing hospital organizations in the UK (Mid Staffordshire; Southern Health). Subsequent reports (e.g. Francis, 2013) recommended the need for a change of culture within the NHS, embodying compassionate and patient centered care that is underpinned by the NHS constitution and values. These values could be seen to reflect the moral obligation of healthcare staff to work in an empathic way with service users.

The clinical relevance of the emotive, cognitive, and behavioral dimensions have been demonstrated empirically with varied emphasis (Decety & Jackson, 2004; Eisenberg & Eggum, 2009; Mercer & Reynolds, 2002). Stepien and Baernstein (2006) discussed how engagement on a solely cognitive level could lead to empathic statements appearing superficial, therefore emotional engagement is necessary to enhance the interaction, building trust within the therapeutic relationship. Here the focus is on the importance of the cognitive and emotional dimensions.

Conversely, service users have reported that a clinician's ability to firstly, understand them (cognitive dimension) and secondly, express this understanding (behavioral dimension), is a key aspect in the therapeutic relationship (Shattell, Starr, & Thomas, 2007). This emphasis on understanding, and the links with developing a meaningful relationship, are supported by Hojat et al. (2002) who highlight how developing a meaningful relationship with service users is contingent on an understanding of their cognitive and affective states. Mercer and Reynolds (2002) also considered ‘understanding’ to be an important facet in responding empathically.

This connection between empathy and relationship with service users has been cited in previous research. Roter et al. (1997) and Suchman, Roter, Green, and Lipkin (1993) found that service users and clinicians felt greater satisfaction with an interaction when there was an increase in empathy. Improved clinical outcomes have also been linked to increased clinician empathy and a good therapeutic relationship (Burns & Nolen-Hoeksema, 1992; Elliott, Bohart, Watson, & Greenburg, 2011; Krupnick et al., 1996). Therefore empathy, irrespective of the particular dimension or definition, could be viewed as an important component of the staff - service user relationship, and subsequently crucial to ensuring the delivery of quality care (Yu & Kirk, 2009).

Yu and Kirk (2009) highlighted the importance of ensuring the measurement of empathy is robust, if it is to be utilized as an outcome for quality of care. In reviewing the measurement tools for empathy in nursing staff they found no ‘gold standard’ tool (Yu & Kirk, 2009). They cited the Empathy Construct Rating Scale ([ECRS]; La Monica, 1981) as the most widely used in the reviewed literature and scored highest on their quality rating scale; however they found that of the 12 measures of empathy they reviewed, none were both psychometrically and conceptually satisfactory. Additionally, the use of service users in the development of the tools was considered lacking and recommended in future research.

### 1.3 Burnout and empathy: is there a relationship?

In addition to improving the psychometric and conceptual measurement of empathy, understanding factors which impact on a clinician's empathic ability is also beneficial. Studies have shown how, despite being an important component in providing effective care, empathy also creates vulnerability for stress related conditions such as compassion fatigue and professional emotional exhaustion (Figley, 2002, Rothschild, 2006). As emotional exhaustion is considered one aspect of the burnout construct, it is not surprising that links have been established between empathy and burnout (Àstrom, Norberg, Nilsson, & Winblad, 1987; Ferri, Guerra, Marcheselli, Cunico, & Di-Lorenzo, 2015). However, findings have been inconclusive in establishing the direction and nature of the relationship (Picard et al., 2015), with empirical evidence demonstrating both a negative and positive correlation between high burnout scores and empathy (Hoffman, 2000; Mercer & Reynolds, 2002).

In an editorial, Zenasni, Boujut, Woerner, and Sultan (2012) proposed three hypotheses for the relationship between burnout and empathy: (1) burnout reduces the ability of clinicians to respond empathically; (2) being empathic draws significantly on personal resources and thus causes burnout; and (3) being empathic protects clinicians from burnout. In their proposal, Zenasni et al. (2012) only summarize the research, providing no empirical evidence for their directional hypotheses. It is important to distinguish that burnout is an occupational stress syndrome, while empathy could be viewed as a human capacity. Although impaired empathy could be a feature of burnout syndrome (hypothesis 1), it is harder to conceptualize that burnout could be a feature of low levels of empathy.

In light of this, it is proposed that the original three hypotheses can be reduced to; 1) There is a negative association between burnout and empathy (as one construct increases the other decreases), and 2) there is a positive association between burnout and empathy (high burnout is associated with high empathy). Zenasni et al.'s (2012) editorial does not constitute a systematic review of the literature; instead it can be seen as a provisional framework for reviewing the literature in the area. A preliminary literature search indicated no existing systematic review exploring the relationship between burnout and empathy.

### 1.4 Rationale and aims

The impact of burnout on staff well-being, and subsequent financial burden on health organizations provides a rationale for understanding the relationship between burnout and empathy. This understanding could serve to inform future research and practice around preventative actions within services. Measures of burnout could be utilized within services to identify ‘at risk’ members of staff at whom these preventative interventions could be targeted. Similarly, as empathy is considered key to clinician service user interactions a greater understanding of the role of burnout in empathic responses may have a positive effect on service user experiences. Ham, Berwick, and Dixon (2016) cite quality of care as the focus of many government policies (Department of Health [DoH], 1998, 2008). Therefore exploration into burnout and empathy in healthcare staff, holds organizational and clinical importance.

The aims of this review were to systematically identify, appraise, and summarize the empirical evidence regarding the relationship between burnout and empathy amongst healthcare workers. Specifically the review considered the following questions:

1. Is there an association between burnout and empathy?
2. What is the relationship between burnout and empathy?
3. To what methodological standard has the current research been conducted, and how does this affect the ability to draw conclusions?

## 2 Method

This review followed the Preferred Reporting Items for Systematic Reviews and Meta-Analyses ([PRISMA]; Liberati et al., 2009) format. In line with this, the methods of the review were specified in advance in a protocol registered on the international prospective register of systematic reviews (www.crd.york.ac.uk/PROSPERO, CRD42015029564).

### 2.1 Information sources

Initial scoping searches were completed to define the search terms: (Burnout OR Burn-out OR “Burn out”) AND (Empathy OR Empath*). Publications were retrieved by searches on five electronic databases: MEDLINE, PsycINFO, CINAHL Plus, PubMed, and SCOPUS. The search was expanded manually by searching reference lists of eligible articles and by citation tracking the selected studies on Web of Science. The databases were searched for studies in the English language, from inception of each journal to February 2017.

### 2.2 Eligibility criteria

The inclusion and exclusion criteria were generated by the primary researcher through preliminary scoping searches of the literature and verified by supervisors. Quantitative non-intervention studies were included in this review. If all other inclusion criteria were met, intervention studies addressing factors which moderate or mediate burnout were included where data was available pertaining to the relationship at baseline, between burnout and empathy. Only studies available as full-text in English were included due to time and budget restrictions. There were no restrictions applied to publication format (e.g. journal article, thesis etc.). Studies that did not provide enough detail to ascertain whether or not they met the inclusion criteria were excluded from the study.

#### 2.2.1 Outcomes

Burnout and empathy were considered outcomes for the purpose of the review, given the unclear relationship between the two variables. For inclusion, studies must have utilized the Maslach Burnout Inventory (MBI; Maslach & Jackson, 1981) to assess burnout and a formal outcome measure to assess empathy (e.g. Interpersonal Reactivity Index [IRI], Davis, 1983). This ensured the construct validity and reliability of the data could be ascertained if available and maximized the homogeneity of the studies in terms of burnout measurement. Studies using translated standardized measures were also included if the study was reported in English.

#### 2.2.2 Participants

Studies were eligible if they reported on participants who had a nursing (health or mental health) or medical professional background, regardless of participant age, ethnicity or nationality. Students or trainees were excluded as their role and pressures are likely to differ from that of a qualified professional, for example, due to the demands placed on them to complete academic aspects of their training. Although burnout is documented to affect many human services, studies recruiting non-healthcare professionals (e.g. teachers, veterinarians) were excluded as the review aimed to address healthcare related literature.

Nurses and doctors are often expected to see a large volume of patients for more limited periods, compared with other professions such as psychology who would typically engage in a therapeutic relationship over a longer period of time. The nature of the relationship between these professionals therefore may differ, with doctors and nurses adopting a more prescriptive didactic stance guiding service users through a medically dominated process. On this basis allied healthcare professionals (e.g. psychologists, therapists, and social workers) were excluded because their roles and relationships with patients are different from that of a nurse or medical doctor. Studies conducted in both adult and paediatric healthcare settings, including mental health services were included.

### 2.3 Search strategy

**Fig. 1:**
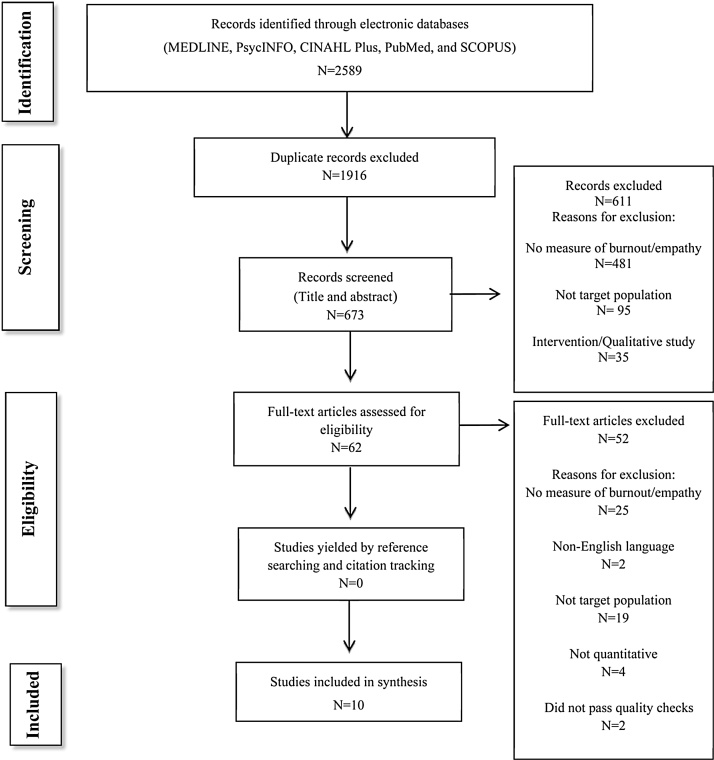
Flow Chart of Literature Search Process.

Titles and abstracts were initially reviewed to check they met the inclusion criteria. A second researcher independently screened a random 10% of these abstracts to check the reliability of the screening process, with 100% agreement between both researchers. Articles not meeting the inclusion criteria were removed (see Fig. 1). Two independent researchers came to 100% agreement when screening the eligible nine articles using the inclusion criteria.

References of eligible articles were searched, however no additional articles were found. All intervention studies that met the other inclusion criteria were screened for baseline relationship data between burnout and empathy, however none of these studies provided this data and were therefore excluded from the review. The process of screening identified publications is reported using the PRISMA diagram (Liberati et al., 2009) (see Fig. 1).

### 2.4 Data extraction

Data was extracted independently by two researchers using a piloted extraction form. Data was extracted pertaining to study characteristics (author, year, country, design, outcome measures, and primary purpose), participant information (number of participants, mean age, gender, job role), and study findings (analysis and outcomes relating to burnout and empathy). The value of the main measure of association between burnout and empathy (total, and where appropriate, subscales) was extracted for each study, together with statistical significance and precision estimates where available.

### 2.5 Methodological quality (risk of bias in individual studies)

A specific quality assessment tool was selected based on the cross sectional design utilized by all of the studies in the review. A search of the literature revealed one quality assessment tool specifically designed for reviewing cross sectional studies. The Agency for Healthcare Research and Quality tool (Williams, Plassman, Burke, Holsinger, & Benjamin, 2010) was adapted for use in this review. The adaptations to the tool included changes to terminology and omission of some items that were not relevant to the constructs of interest, as in previous studies which have utilized this tool (Taylor, Hutton, & Wood, 2015).

Categories for assessment included: sample selection, size, and description; validation of outcome measurements for empathy and burnout; analysis of confounders; and handling of missing data. Studies were assessed using four categories, as having ‘met’, ‘not met’, ‘partially met’, or ‘unable to ascertain’ if they met the quality criteria. A total (numerical) quality score was not assigned to the individual studies, as evidence demonstrates this does not provide a better quality systematic review (Jüni, Witschi, Bloch, & Egger, 1999). To date this tool does not have any reported reliability or validity data. Its construction is cited by the authors (Williams et al., 2010) to be based on quality criteria utilized in two previous evidence reports by the Agency for Healthcare Research and Quality (Myers et al., 2008, Wang et al., 2004). Two researchers completed the quality checks independently, following which a Kappa score was calculated to establish reliability of the decisions based on the tool. Any discrepancies were resolved by discussion with supervisors (see Table 2).

**Table 1:** Data Extracted from Studies Pertaining to Study Characteristics, Participant Details, and Main Findings.

Study Characteristics				Participant Characteristics						Study Results			
	Authors, Year, Country	Setting/Specialty	Measures	N=	Profession			Gender	Age (years)	Relationship to Empathy			
			Empathy		Nurses	Medics	Other			EE	DP	PA	Other
A	Baxter (1992); America	Acute care hospital setting	BLRI	124	√			M = 5	Mean = 38.9	*r* = −0.14 ^e^	*r* = −0.33^a^	*r* = +0.21^d^	
								F = 119	(SD 8.9)				

B	Bradley (1995); America	Adolescent medical unit, Emergency department, Adolescent psychiatric unit	EES	79	√		√	M = 12	Mean = 35.7	*r* = −0.07^d^	*r* = −0.15^d^	r = −0.01^d^	
								F = 67	(SD 5.9)				

C	Kellner (2001); America	Emergency Services	EES	124	√	√		M = 55	Mean = 38	*r* = +0.40^a^	*r* = +0.24^c^	*r* = −0.25^c^	
								F = 69	(SD 11.5)				

D	Lamothe et al. (2014); France	Primary Care-GP practices	Emotional Empathy (Empathic Concern) – TEQ	294		√		M = 151	Mean (M) = 53.5	Cognitive & Emotional Empathy & Burnout Subscales EE:	Cognitive & Emotional Empathy & Burnout Subscale DP:	Cognitive & Emotional Empathy & Burnout Subscales PA:	*r* = −0.24^c^ Total Burnout Score & Reduced Cognitive Empathy
			Cognitive Empathy – JSPE (Perspective Taking Subscale)					F = 143	(SD 8.6)	*r* = not reported	*r* = −0.18 to −0.32^c^	*r* = +0.18 to +0.40	*r* = −0.17^c^ Total Burnout Score & Reduced Emotional Empathy
									Mean (F) = 48.3				Linear Regression (cognitive and emotional empathy interaction as predictors): Higher emotional empathy (β = −0.17^d^) & cognitive empathy (β = −0.21^a^) predicted lower burnout.
									(SD 9.4)				

E	Lee et al. (2003); Korea	Tertiary hospitals	Emotional Empathy – EES	178	√			F = 178	Mean = 30	Correlations	Correlations	Correlations	
			Cognitive Empathy – BLES							Cognitive Empathy & Burnout Subscales EE:	Cognitive Empathy & Burnout Subscales DP:	Cognitive Empathy & Burnout Subscales PA:	
										*r* = −0.25^a^	*r* = −0.36^a^	*r* = +0.47^a^	
										Emotional Empathy & Burnout Subscales:	Emotional Empathy & Burnout Subscales:	Emotional Empathy & Burnout Subscales:	
										*r* = −0.03	*r* = +0.03	*r* = −0.07	
										Hierarchical Regressions:	Hierarchical Regressions:	Hierarchical Regressions:	
										Burnout subcategories and Cognitive empathy:	Burnout subcategories and Cognitive empathy:	Burnout subcategories and Cognitive empathy:	
										β = −0.15^e^	β= −0.24^b^	β = +0.27^a^	
										Burnout subcategories and Emotional empathy:	Burnout subcategories and Emotional empathy:	Burnout subcategories and Emotional empathy:	
										β = −0.02^e^	β = −0.01^e^	β = 0.00^e^	

F	Tei et al. (2014); Japan	Hospital	IRI	25	√			M = 5	Mean = 26				Correlations of Burnout Subscale:
								F = 20	(SD 3.14)				Depersonalization and Empathy Subscales;
													*r* = +0.39 Perspective Taking
													*r* = −0.02 Empathic Concern
													*r* = −0.10 Personal Distress
													Correlations of Burnout Subscale:
													Emotional Exhaustion & Empathy Subscales
													*r* = +0.51^c^ Perspective Taking
													*r* = +0.14 Empathic Concern
													**r* = +0.24 Personal Distress*
													

G	Torres et al. (2015); Spain	Primary Care-GP practices	JSPE	108		√		M = 39	not given				high empathy and low burnout, no inferential statistics reported
								F = 69					

H	Walocha et al. (2013); Poland	Hospitals, Outpatient clinics, university departments	EES, TAT	71		√	√	M = 46	Range = 25–68	Empathy and EE subscale of Burnout	Empathy and DP subscale of Burnout	Empathy and PA subscale of Burnout	Spearman's Correlation Co-Efficient:
								F = 25		G1	G1	G1	Whole Sample;
										*r* = −0.01	*r* = −0.13	*r* = +0.18	*r* = −0.23^d^ Low Personal Accomplishment & Empathy
										G2	G2	G2	
										*r* = −0.13	*r* = −0.37^e^	*r* = +0.11	
										G3	G3	G3	
										*r* = −0.34^e^	*r* = −0.39^d^	*r* = +0.02	

I	Ferri et al. (2015); Italy	General Hospitals, surgical & medical wards	BEES	162				M = 32	Mean = 39	r = −0.245	Not statistically significant (no figures recorded)	r = 0.266	
								F = 130	SD = 9				

J	Yuguero et al. (2017); Spain	Primary Care, urban & rural GP practices.	JSPE	267	√	√		M = 58	Median = 48	r = −0.1	r = -0.2^a^	r = 0. 3^a^	Overall MBI/JSPE: r = −0.2^a^
								F = 209	Range 31–65				

Note: p < 0.001^a^, p < 0.005^b^, p < 0.01^c^, p < 0.05^d^, p > 0.05^e^

Measures: Maslach Burnout Inventory ([MBI], Maslach & Jackson, 1981); Barrett-Lennard Relationship Inventory ([BLRI], Barrett-Lennard, 1962); Mehrabian Emotional Empathic Tendency Scale ([EES], Mehrabian & Epstein, 1972), Toronto Empathy Questionnaire ([TEQ], Spreng, McKinnon, Mar, & Levine, 2009), Jefferson Scale of Physician Empathy ([JSPE], Hojat et al., 2001); Barrett-Lennard Empathy Scale ([BLES], Barrett-Lennard, 1962); Interpersonal Reactivity Index ([IRI], Davis, 1983); Thematic Apperception Test ([TAT], Murray, 1951); Balanced Emotional Empathy Scale ([BEES], Meneghini et al., 2006).

Burnout and Empathy Results: (G1) Surgical, (G2) Non-surgical, (G3) Primary Care.

**Table 2:** Agreed Outcome of Quality Assessment of Study Methodology.

		Unbiased selection of participants	Sample size	Adequate description of the cohort	Validated method for measuring burnout	Validated method for assessing empathy	Response rate	Analysis controls for confounding	Analytic methods appropriate
A	Baxter (1992)	Yes	Yes	Yes	Yes	Yes	Yes	Yes	Yes
B.	Bradley (1995)	Yes	Yes	Yes	Yes	Yes	No	Yes	Yes
C	Kellner (2001)	Partially	Partially	Yes	Yes	Yes	Partially*	Yes	Yes
D	Lamothe et al. (2014)	Yes	No	Yes	Yes	Yes	Yes	Yes	Yes
E	Lee et al. (2003)	Yes	No	Yes	Yes	Yes	Yes	Yes	Yes
F	Tei et al. (2014)	Yes	No	Yes	Partially	Partially	Yes	Yes	Yes
G	Torres et al. (2015)	Yes	No	Yes	Partially*	Partially	Yes	Yes	Yes
H	Walocha et al. (2013)	No	No	Partially	Can't tell	Can't tell	No	No	Yes
I	Ferri et al. (2015)	Partially	No	Yes	Yes	Yes	Yes		Yes
J	Yuguero et al. (2017)	Partially*	No	Yes	Yes	Yes	Yes		Yes

Note: * Identifies initial scoring variations between researchers.

### 2.6 Data synthesis and analysis

Results tables (see Table 1) were used to capture the extracted data and quality assessment process for each study individually and the findings were narratively synthesized across studies.

## 3 Results

### 3.1 Study selection

Ten articles were included in the review (see Fig. 1). No additional papers were found by hand searching the reference lists of eligible articles.

### 3.2 Study characteristics

Study characteristics are reported in Table 1. All studies utilized a cross sectional design and were published between 1990 and 2017. The studies were conducted in primary and secondary care health settings. Three studies [D, G, J] recruited participants within Primary Care General Practices, whilst three [A, E, F] of the studies identified hospitals as their recruitment setting, with an additional study [C] specifically stipulating ‘Emergency Departments’ as their place for recruitment. Three of the studies [B, H, I] reported collecting data across multiple services including acute and outpatient departments.

The studies were all conducted in developed countries with three [A, B, C] carried out in the U.S.A. One study [B] used only the depersonalization subscale of the measure. Seven studies [D, E, F, G, H, I, J] were conducted in countries where English is not the first language (Japan, Spain, Poland, France, Korea, and Italy). Only two of these studies [G, J] stated that they had utilized a translated (Spanish) version of the MBI, referencing empirical validation.

In contrast, the construct of empathy was measured utilizing a wide variety of validated measures. The Mehrabian Emotional Empathy Scale ([EES], Mehrabian & Epstein, 1972) was utilized by four studies [B, C, E, H]. One of these studies [E] also used the Barrett-Lennard Relationship Inventory (BLRI, Barrett-Lennard, 1962) to measure cognitive empathy (see Table 1). Studies D and E were the only ones to delineate the measurement of cognitive and emotional aspects of empathy with separate measures. The following empathy scales were used in one study only: the Interpersonal Reactivity Index [F] (IRI, Davis, 1983); the Empathy Construct Rating Scale [G] (ECRS, La Monica, 1981); the Balanced Emotional Empathy Scale [I] (BEES, Meneghini, Sartori, & Cunico, 2006) and the Jefferson Scale of Physician Empathy [J] (JESPE, Hojat et al., 2001). Seven of the studies focused on burnout and empathy exclusively [A, B, C, G, H, I, J] however other constructs including spirituality, empowerment, emotional dissonance, sick leave prescribing, coping styles, and attitudes towards patients with dementia were included within the other studies [D, E, F].

### 3.3 Participant details

Different terminology was utilized for reporting participant profession, without clarification of the job role. Therefore some of the participants may have had the same job role but under different job titles, although it was not possible to ascertain this from the information provided by the authors. This may be accounted for by the variety of countries the studies were conducted in.

Four of the studies [A, E, F, I] cite ‘Nurses’ as the profession of all of their participants, with one study [A] specifying ‘Registered Nurses’. One study [B] reported recruiting Mental Health Workers in addition to Registered Nurses. Taken together over half of the studies conducted their research with a target population of ‘nursing professionals’.

Two of the remaining studies [D, G] reported recruiting medical doctors exclusively and one study [H] recruited participants who came from different medical specialties, including non-surgical and surgical medics and primary care physicians. Two studies [C, J] had a mixed sample of nurses and physicians.

Nine of the studies [A, B, C, D, F, G, H, I, J] recruited both male and female participants and all of these studies reported over 50% of their mixed sample as female. Two studies [D, H] conducted with medical doctors, reported more male than female participants. Study [D] reported only 2% difference in the gender of their sample, in favor of male participants. One study [E] reported that all of their participants were female, they did not indicate that this was an inclusion criteria. Six [A, D, E, G, I, J] of the ten studies reported a participant response rate. These varied from 39% to 81%. Seven studies [A, B, C, D, E, F, I] reported the mean age of their samples and across these studies the mean ranged from 26 to 48 years. Studies H and J reported the range of their participants. One study [G] did not report participant age (see Table 1).

### 3.4 Risk of bias within studies

The assessment of methodological quality is presented in Table 2. The most common methodological problem related to sample size. Seven studies failed to provide a power calculation to justify or contextualize their sample size [D, E, F, G, H, I, J]. This could indicate that analysis of the correlation between burnout and empathy may have been underpowered, which could lead to inflated Type II error. However it was not possible to establish if the studies were underpowered or if the authors had failed to report an a priori sample size calculation.

Study [H] scored least favorably, with a rating of ‘no’ or ‘can’t tell’ across six of the eight criteria in the assessment tool. All of the studies utilized self-report measures of burnout and empathy. Two studies [G, J], reported translating one of the measures into the language of the participants in the study, however there were a further five studies [D, E, F, H, I] that were conducted in countries where English is not the first language. These studies may have utilized translated measures but failed to report this information.

### 3.5 Reporting of results in individual studies

All of the studies reported correlational analyses of their data (see Table 1). Two of the studies [D, E] also conducted linear regressions. All of the studies that reported the correlation between empathy and the separate subscales DP, PA and EE of burnout. However one of those [G] did not report any inferential statistics; instead only narratively describing the type of correlation that had been found for one of the subscales. One study [I] did not report statistics for the DP subscale only.

Two studies [D, E] defined two aspects of empathy (cognitive and emotional), utilizing different measures for each. A third study [H] also measured behavioral components of empathy through the subscale of an empathy measure. Three studies [G, I, J] reported empathy as a total score. Study [F], which administered the IRI (Davis, 1981), reported the burnout subscales in relation to the empathy subscales.

### 3.6 Evidence for hypothesis one: negative association between burnout and empathy

Eight studies’ findings clearly supported this hypothesis [A, B, D, E, G, H, I, J]. Study [H] demonstrated findings that supported this hypothesis across all three of their participant sub-groups (Primary Care Physicians, Non-Surgical Specialists, and Surgical Specialists), with differing strengths of correlation. They reported a moderate negative correlation between DP and empathy for Non-Surgical and Primary Care doctors (see Table 1). A moderate negative correlation for EE and empathy was only found within the Primary Care doctors. These results should be interpreted with caution as the quality assessment was weak.

Study [D] reported a weak to moderate, negative correlation between DP and empathy, however no *r* values for the EE subscale of the MBI were given. PA was positively correlated with empathy (see Table 1). A separate score for cognitive and emotional empathy in relation to a total score for burnout was reported. Cognitive empathy was negatively correlated with total burnout score and emotional empathy had a weak, but significant, negative correlation with total burnout score (see Table 1).

Study [E] found no significant correlation between emotional empathy and burnout, however their results supported study [H] reporting a moderate correlation between cognitive empathy and DP (see Table 1). Findings for EE and cognitive empathy also support [H] with a weak negative correlation. A strong positive correlation was reported between PA and cognitive empathy. Study [A] found a positive correlation between PA and empathy supporting the above studies. The findings for DP and EE subscales were also in support of [E, H] with negative correlations reported.

Study [G] reported no inferential statistics, however descriptive data suggested that of the participants who scored high on empathy, more scored lower on burnout (72.1%). The sample size for the professionals who reported high burnout was very small (n = 7) when compared with the number of participants who reported low burnout and high empathy (n = 60), this implies that there may be a low statistical power to detect small effects. Study [B] reported no correlation between empathy and the PA and EE subscales of the MBI. However DP was negatively correlated with empathy, providing some evidence for hypothesis one.

Study [I] reported a highly significant negative correlation between the Balanced Emotional Empathy Scale (BEES) and the EE subscale and a highly significant positive correlation between the BEES and the PA subscale. The relationship between the DP subscale and the BEES score was not statistically significant and the direction of the relationship is not reported.

Study [J] found a significant negative relationship between the Jefferson Scale of Physician Empathy (JSPE) and both the MBI total score and the DP subscale. There was a significant positive relationship between the JSPE and the PA subscale. A weak negative correlation between the JSPE and the EE subscale was not statistically significant.

Despite there being seven studies that provided evidence for this hypothesis there is variation in the strength of the correlations and level of significance of the findings that are reported. Due to some of the poor reporting quality from one study [G] it has not been possible to fully synthesize and compare those findings. In summary, the evidence for this hypothesis appears to be complex and nuanced.

### 3.7 Evidence for hypothesis two: a positive correlation between burnout and empathy

Study [C] was the only study to provide consistent support for this hypothesis. Statistically significant, weak positive and moderate to strong positive correlations with empathy were found for DP and EE respectively (see Table 1). The small *p* value reported indicates strong evidence to reject the null hypothesis that there is no association between empathy and burnout. PA was found to have a weak negative correlation with empathy. The quality checks completed on this study indicated that across all of the domains the study provided at least partial information to fulfill the criteria, this indicates that the standard of reporting and quality of the study was adequate. As part of this, the study provided a power calculation, indicating that the number of participants recruited (n = 124) was less than the minimum required to ensure adequate power (n = 140).

Alongside support for hypothesis one, study [F] also provided support for hypothesis two. The results indicated that all subscales on the IRI (PT, EC, PD) had strong to moderate, positive correlations with the EE subscale of the MBI (Maslach & Jackson, 1981) (see Table 1). This concurs with study [C] indicating that those clinicians with higher empathy scored higher on the EE subscale of the MBI (see Table 1). In their discussion, study [F] concluded that their results supported the ‘compassion fatigue’ theory, whereby clinicians who demonstrate high levels of empathy suffer from compassion fatigue, which then leads to burnout. However they found a weak negative correlation between two subscales of the IRI (PD, EC) and DP, which could be seen to support hypothesis one. As study F provided support for both hypotheses, this could be seen as somewhat contradictory. This could be explained by the small sample size (n = 11) which is indicative of an underpowered study. The result must therefore be viewed with caution. These negative correlations would provide support for the first hypothesis and therefore contradicts the positive correlations reported between the EE subscale and empathy (see Table 1).

In contrast to the overwhelming support for hypothesis one, there was less evidence found in support of hypothesis two, with only one study providing consistent support for this hypothesis across their findings. The second study discussed in relation to this hypothesis [F] found aspects of their results to support both hypotheses. It would appear therefore that within the studies reviewed there is more support for a negative association between empathy and burnout.

## 4 Discussion

This review sought to explore the current literature conducted with medical doctors and nurses to explore the relationship between burnout and empathy.

In addressing the first question of this review, whether there is an association between burnout and empathy, evidence was found in all of the studies included in this review to support the previously suggested association between burnout and empathy (Àstrom et al., 1987; Ferri et al., 2015; Miller, Stiff, & Ellis, 1988). These two distinct constructs which are so central to effective healthcare delivery appeared to be related. However, the size and statistical significance of the reported correlations varied. Only three studies [C, E, H] reported large correlations, as defined using Cohen's criteria for behavioral sciences (Cohen, 1992). This reflects previous research in the area which has reported varying strengths of correlation (Hoffman, 2000; Mercer & Reynolds, 2002).

As highlighted in a previous editorial (Zenasni et al., 2012), findings relating to the direction (positive/negative) of the relationship between burnout and empathy were not unanimous. The second aim of this review was to explore the ambiguous relationship between burnout and empathy within the framework of two opposing hypotheses: 1) there is a negative association between burnout and empathy, (as one construct increases the other decreases), and 2) there is a positive association between burnout and empathy (high burnout is associated with high empathy). Taking into consideration the methodological rigor, homogeneity in terms of MBI (Maslach & Jackson, 1981) usage, number of concurring findings, and the strength of the correlations reported, the current review found the strongest evidence for the first hypothesis that burnout and empathy were negatively correlated, inferring that as the presence of one construct increases the other decreases.

Eight of the ten studies reported a negative relationship between burnout and empathy supporting the first hypothesis. As these studies were cross sectional it is not possible to infer causality. However, despite this, some of the studies discussed their findings in relation to high burnout causing low empathy. It is important to be cautious with these statements, as the research design does not allow for a definitive statement; instead these could be viewed as potential hypotheses that could be explored in future research.

The studies supporting hypothesis one were conducted within heterogeneous settings (e.g. outpatient departments, nursing home, emergency department), involving participants from different professions (e.g. registered nurses, general practitioners, surgeons). This could be seen to demonstrate that the association between empathy and burnout is consistent across these settings within these populations and therefore is relevant to all healthcare professionals. This would therefore support the need for intervention and awareness across all staff groups at an organizational level. It is important to note however, that transferring findings between contexts should be done with caution as these environments are diverse and unique.

Two of these studies satisfied all of the quality assessment criteria indicating that reliability of the findings is high. However seven of the studies failed to report enough data pertaining to their sample size. This makes it difficult to ascertain if their studies were underpowered. One of the studies reported moderate correlations in support of this hypothesis however the quality assessment rating indicated that 50% of the domains were given a rating of ‘no’ (see Table 2). This indicated that the quality of the reporting or design was not adequate. Therefore this may affect the reliability of the findings.

There was only one study which provided support for the second hypothesis, of a positive correlation between burnout and empathy. This hypothesis maps on to the suggestions of Maslach and Jackson (1981), that those staff who are empathic will become burnt out.

The evidence found by this review supports burnout as a cross-cultural construct. The studies were conducted in a variety of countries that represented several continents (Asia, North America, and Europe). Whilst this can be interpreted as a strength of this review, it is important to note that of the seven studies that were conducted in countries where English was not the first language, only two reported information about the translation of measures. Evidence suggests that the language of a questionnaire can affect the way a participant responds (Harzing & Maznevski, 2002). Therefore researchers should systematically establish equivalent terms in their adapted measures (Mullen, 1995).

### 4.1 Clinical implications

The predominant finding of this review was the largely consistent support for a negative relationship between burnout and empathy amongst healthcare staff (e.g. high burnout - low empathy). The evidence in the literature highlights the prevalence of burnout within healthcare staff and possible consequences on quality of care (Poghosyan et al., 2010) and staff attrition (Maslach, 2003). Therefore, measuring levels of burnout in staff could be utilized as a way of identifying and targeting staff who are ‘at risk’ of developing burnout. They could then be offered preventative interventions. For example, in a recent evidence review for Public Health England, Bagnall, Jones, Akter, and Woodall (2016) provided an overview of the prevention and intervention literature on burnout and work-related stress in individuals and within organizations. They found that interventions to prevent or reduce burnout were usually aimed at an individual level including staff training, workshops, and cognitive-behavioral programs. A greater understanding of burnout in terms of treatment and prevention is highlighted as being important from a public health and organizational perspective in the context of reducing absenteeism and increasing productivity (Bagnall et al., 2016).

If the impact of burnout on staff cannot be reduced, then interventions to increase/sustain empathy within staff groups, and perhaps therefore guard against burnout, may be useful. This is particularly relevant given the links demonstrated in the literature between burnout, empathy and quality of care (Brockhouse et al., 2011, Poghosyan et al., 2010). One potential mechanism of this may be through the use of psychological formulation, as increasing clinician understanding of service users is often seen as integral to the development and maintenance of empathic interactions (Yu & Kirk, 2009). Future research could therefore seek to explore the utility of psychological formulation in increasing empathy.

### 4.2 Strengths and limitations of the review

The current review has followed a predetermined protocol and was informed by the PRISMA guidelines (Liberati et al., 2009) to ensure methodological rigor. However the authors acknowledge that it has a number of limitations which should be considered when interpreting the conclusions.

The current review excluded studies that were not available in English due to time and budgetary restrictions, which would not allow for translation of articles. Given that seven of the ten studies were conducted in countries where the first language was not English, it could be reasonably assumed that there may be other relevant studies that have been conducted and are published in languages other than English. The implications of this on the current review are that it may not have captured all of the current research looking at the relationship between empathy and burnout. Therefore the reliability of the conclusions may be affected. However by including studies where there is an English translation of the article available, the current review has avoided an English-speaking bias that can be seen in some literatures e.g. violence (Whittington et al., 2013).

In addition, this review excluded papers that used qualitative or mixed methodology as it was felt comparison between studies which utilized standardized psychometric assessments to measure the constructs would be more reliable. However qualitative studies provide a richness of data that is lost in the numerical values assigned in standardized measures. This more descriptive data could provide greater insight into the experiences of staff relating to burnout and responding empathically, and subsequently the relationship between these two constructs.

### 4.3 Suggestions for future research

As previously highlighted, all of the studies included in this review utilize a cross-sectional design. This is due in part to the exclusion of intervention studies, however intervention studies were screened for inclusion if they provided baseline data. Whilst the review has established useful findings as to the association between empathy and burnout, it has not been possible to progress further in commenting on the existence or direction of causality of this association. Many of the authors in the included studies recognize this limitation, highlighting the need for future research to adopt a longitudinal causational design in order to begin to address this gap in the literature. However the author acknowledges that longitudinal research is not without difficulties, as retention of participants can be challenging and affect the viability of the research.

Whilst the inclusion criteria of the current review restricted the profession of participants included in the study, it was noted that there are currently no studies based in forensic settings investigating the relationship between empathy and burnout. This setting may be of particular interest, as societal norms would suggest that being empathic to those with a forensic record might be more difficult (Sandhu, Rose, Rosthill-Brookes, & Thrift, 2012), and working in this environment where there is an increased risk of physical violence and verbal aggression may put staff at greater risk of burnout (Joseph, 1993).

Despite extensive research in this area no previous systematic review with this aim was identified prior to commencing the current review. This review has made some progress in outlining the state of the current research investigating burnout and empathy within nurses and medical doctors. Effect sizes have been reported to provide some statistical indication of the strength of the findings, although not empirically tested. However, future research could build on this by completing a more detailed meta-analysis of the data.

Although all of the studies included in this review approach empathy and burnout as distinct constructs, it could be suggested EE and PA are more distinct from empathy, while DP and a lack of empathy overlap. Therefore it is likely that these constructs would be correlated. Future research may wish to explore the individual constructs of empathy and burnout to develop this further. This future research would be aided by the development of improved psychometric measurement of clinician empathy. This could help capture empathy more accurately. In addition to supporting further research into the distinction between empathy and burnout, development of an improved psychometric measure could also help to inform future research and enhance development of ‘empathy-enhancing’ interventions and training. Measurement of empathy could also serve a purpose within staff recruitment in line with the NHS constitution and values based recruitment.

Finally, the results support previous research in emphasizing the importance of decreasing burnout in care staff, and the potential for increasing levels of empathy as a way of doing this. Further research exploring mechanisms by which empathy can be increased, and any resulting impact on levels of burnout, would therefore be beneficial.

## Conflict of interest

None.

## Funding

this research did not receive any specific grant from funding agencies in the public, commercial or not-for-profit sectors.
